# Algorithm for UAV path planning in high obstacle density environments: RFA-star

**DOI:** 10.3389/fpls.2024.1391628

**Published:** 2024-10-17

**Authors:** Weijian Zhang, Jian Li, Weilin Yu, Peng Ding, Jiawei Wang, Xuen Zhang

**Affiliations:** ^1^ College of Information Technology, Jilin Agricultural University, Changchun, China; ^2^ Bioinformatics Research Center of Jilin Province, Changchun, China

**Keywords:** precision agriculture, RFA-star algorithm, plant protection UAV, feature attention mechanism, path planning

## Abstract

Path planning is one of the key elements for achieving rapid and stable flight when unmanned aerial vehicles (UAVs) are conducting monitoring and inspection tasks at ultra-low altitudes or in orchard environments. It involves finding the optimal and safe route between a given starting point and a target point. Achieving rapid and stable flight in complex environments is paramount. In environments characterized by high-density obstacles, the stability of UAVs remains a focal point in the research of path planning algorithms. This study, utilizing a feature attention mechanism, systematically identifies distinctive points on the obstacles, leading to the development of the RFA-Star (R5DOS Feature Attention A-star) path planning algorithm. In MATLAB, random maps were generated to assess the performance of the RFA-Star algorithm. The analysis focused on evaluating the effectiveness of the RFA-Star algorithm under varying obstacle density conditions and different map sizes. Additionally, comparative analyses juxtaposed the performance of the RFA-Star algorithm against three other algorithms. Experimental results indicate that the RFA-Star algorithm demonstrates the shortest computation time, approximately 84%-94% faster than the RJA-Star algorithm and 51%-96% faster than the Improved A-Star. The flight distance is comparable to the RJA-Star algorithm, with slightly more searched nodes. Considering these factors collectively, the RFA-Star algorithm exhibits a relatively superior balance between computational efficiency and path quality. It consistently demonstrates efficient and stable performance across diverse complex environments. However, for comprehensive performance enhancement, further optimization is necessary.

## 1 Introduction

Over the past few years, with continuous advancements in science, technology, and productivity, UAV have successfully integrated into various industries (Fang et al., 2023b). Especially in agriculture and forestry (de Castro et al., 2021; Jurado et al., 2022; Raparelli and Bajocco, 2019), geological exploration (Giordan et al., 2020; Ren et al., 2019), wildfire detection (Bouguettaya et al., 2022; Ghali et al., 2022), disaster rescue (Wang et al., 2019), and military (Xiaoning, 2020), the use of UAVs is increasingly widespread, including the study of single UAVs or UAV formations (Fang et al., 2023a; Fang and Xie, 2023). As a crucial component of precision agriculture, crop inspection and monitoring research is of significant importance (Castro et al., 2023). Utilizing various sensors to acquire diverse plant characteristics provides a key information foundation for real-time or future decision-making in plant management (Su et al., 2023; Zhang et al., 2022). UAVs equipped with various sensors can capture multiple crop features, which are used to monitor planting areas and crop growth conditions, assess biological and physical characteristics, predict yields, and detect stress levels. UAV-based crop monitoring has become a critical tool for aiding agricultural producers and improving agricultural management (Gao et al., 2023; Li et al., 2024; Su et al., 2023). However, UAVs often encounter various obstacles during ultra-low-altitude flights for crop monitoring (Wang et al., 2022; Zhu et al., 2023). In environments such as orchards, where plant inspection and monitoring occur, trees and flocks of birds are the primary obstacles for UAVs (Ghaddar and Merei, 2020; Yu et al., 2022). UAVs often operate in environments characterized by high-density obstacles, especially in orchards. Consequently, a key challenge in UAV technology is how to adeptly navigate around these hindrances during task execution. To tackle this issue, numerous researchers have delved into a variety of path planning, formation control, and obstacle avoidance algorithms (Fang et al., 2020b, 2020a). Encompass ant colony algorithms (Gao et al., 2021), Dijkstra's algorithm (Dhulkefl and Durdu, 2019), A-star algorithm (Cai et al., 2019), and artificial potential field methods (Pan et al., 2021), among others. The A-star algorithm has gained widespread usage due to its simple principles and computational convenience (Zhang et al., 2021). However, traditional A-star algorithms exhibit certain limitations. The A-star algorithm necessitates traversing a substantial number of nodes, leading to computational complexity and inefficient pathfinding. As the map area expands, the computational load experiences an exponential growth (Wang and Sun, 2023). In response to these challenges, scholars both domestically and internationally have undertaken extensive research endeavors aimed at optimizing and enhancing these algorithms.

In the realm of A-star algorithm improvement, Zhang et al. introduced a global A-Star path planning algorithm, enhancing the A-Star algorithm based on a bidirectional search strategy. This innovative approach successfully achieved a significant improvement in computational speed, ranging from 47.6% to 52.4%, while substantially reducing the number of traversed nodes by 68.2% to 75.4% (Zhang et al., 2023). Shang et al., utilizing key points around obstacles, introduced a variable step-length A-star to reduce the algorithm's computation time (Erke et al., 2020). He et al. addressed the multi-ship encounter problem in complex scenarios by proposing a dynamic collision-avoidance A-star algorithm. This algorithm is designed to prevent collisions in the presence of known moving obstacles (He et al., 2022). Mandloi et al. introduced a time cost function to overcome computational challenges of A-star in three-dimensional space (Mandloi et al., 2021). By integrating and enhancing the A-star algorithm, To better align the path planning of unmanned aerial vehicles (UAVs) with real operational scenarios, Zhang et al. combined A-star with artificial potential field methods and improvement (Zhang et al., 2021). Rostami improved the repulsive function in the artificial potential field method by introducing an adjustment factor (Rostami et al., 2019). In order to enhance the flexibility of unmanned surface vessels and alleviate computational burdens, Yan et al. integrated virtual structures with the artificial potential field method (Yan et al., 2021). In scenarios characterized by high obstacle density, Andriy et al. proposed a decentralized algorithm designed to manage UAV swarms in environments with high obstacle density. This approach integrates local planning loops with bio-inspired swarm rules to guide the compact UAV swarm within the operational workspace without relying on external infrastructure. By introducing a specially designed on-board UVDAR system, mutual localization among team members is achieved around each UAV, ensuring the stability and coherence of the entire swarm (Dmytruk et al., 2021). Ahmad et al. presented a fully decentralized bio-inspired control method that relies solely on on-board sensor data to safely organize UAV swarms in the environment without the need for communication with other agents. The feasibility and performance of the proposed method were validated and assessed through multiple experiments in both a realistic robot simulator and a natural forest setting (Ahmad et al., 2021).

However, the aforementioned UAV studies primarily focus on flights above the obstacle space (Radoglou-Grammatikis et al., 2020). Although this simplifies operations, it significantly limits the scope of measurements. Relying solely on overhead data makes it difficult to accurately assess the size and health of individual fruits or measure tree diameters. UAVs capable of flying beneath the canopy can overcome these limitations by achieving a good balance between coverage and sensor resolution. Nevertheless, developing a UAV system that can fly at multiple altitudes in large-scale environments and autonomously navigate between tree rows or even beneath the canopy remains a significant challenge (Liu et al., 2022). Therefore, one of the core issues for UAVs flying beneath the canopy is how to effectively avoid these high-density obstacles during task execution. The ability to navigate to a predetermined destination while avoiding obstacles in the path is a fundamental element of autonomous flight. However, UAVs operating at low altitudes often encounter unexpected obstacles, requiring an obstacle avoidance system that is both quick and effective. This often leads to a reduction in operating speed, necessitating a specially designed obstacle avoidance system to ensure safety (Butt et al., 2024). In scenarios characterized by high-density obstacles, the computational time and complexity of the A-star significantly escalates in 3D environments, potentially hindering the smooth attainment of the target position. To address this issue, this study, grounded in a three-dimensional context, introduces an improved algorithm named RFA-Star (R5DOS Feature Attention A-star plus). Leveraging the R5DOS(Regions Connection Calculus-5 Direction Octant Strongly-exists) model for the abstract representation of simple objects, this algorithm incorporates a feature attention mechanism around obstacles based on the perceived obstacle information by the UAV, the RFA-Star algorithm is employed for obstacle avoidance. Built upon the matrix representation of the R5DOS model and integrating a feature attention mechanism, this model enhances the search efficiency of the A-star algorithm in complex environments. It addresses the challenge of safely navigating through high-density obstacles, thereby averting potential safety issues.

The main emphasis of this research centers on enhancing the A-star within three-dimensional settings by employing a topological relationship matrix. The efficacy of the RFA-Star algorithm in environments with high obstacle density and its enhanced capabilities are substantiated through simulation experiments based on models. The subsequent section delineates the contributions and novel aspects presented in this manuscript.

1. Addressing the path planning challenges in complex environments with high obstacle density, such as UAVs needing to inspect and monitor plant information beneath the canopy in orchards, this study improves the A-Star algorithm based on a spatial topological relationship model and proposes the RFA-Star algorithm.
2. Upon detecting obstacles, the RFA-Star algorithm selectively searches for feature points, reducing interference from irrelevant obstacles to the UAV.
3. In scenarios with complex and high obstacle density maps, the RFA-Star algorithm incorporates an improved local A-star algorithm and a feature attention mechanism to guide the UAV successfully around obstacles.

This paper’s organization is structured as follows: Section 2 presents a detailed elaboration of improvements to the A-star and the overarching architecture of the RFA-Star algorithm. In Section 3, the effectiveness of the RFA-Star in path planning is comprehensively validated through simulation and comparative experiments. Section 4 delves into a comprehensive discussion of the experimental results and scrutinizes the limitations of the RFA-Star algorithm. The concluding Section 5 encapsulates the findings of this study and probes potential future research directions.

## 2 Materials and methods

### 2.1 Abstraction of UAV

**Figure 1:**
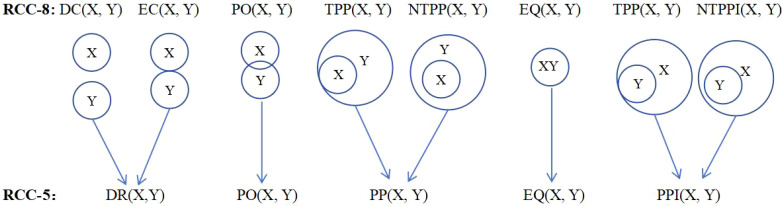
Mathematical expressions and abstract representations corresponding to topological relations.

Li et al. proposed an R5DOS model based on the region connection calculus (RCC) theory in 2020, demonstrating the model's completeness and mutual exclusivity, the model can represent 11,038 possible topological directional relationships among three simple regions in three-dimensional space. Subsequently, they improved and applied it in UAV swarm algorithms and UAV path planning algorithms, providing detailed insights into the improvements made to the R5DOS model (Li et al., 2020, 2022, 2023). This study adopts the improved R5DOS model to define UAVs and obstacles, dividing UAVs into the body region and detection region. The UAV's body region represents the area that ensures absolute safety during flight, while the detection region is responsible for detecting obstacles and target points. As indicated by reference (Li et al., 2022), there are five types of topological relations: Discrete (DR), Partial Overlap (PO), Proper Part (PP), Equal (EQ), and Proper Part Inverses (PPI). The corresponding topological situations are illustrated in **Figure 1**. Among them, RCC-8 is a boundary-sensitive topological relation model (Jonsson et al., 2021).

**Figure 2:**
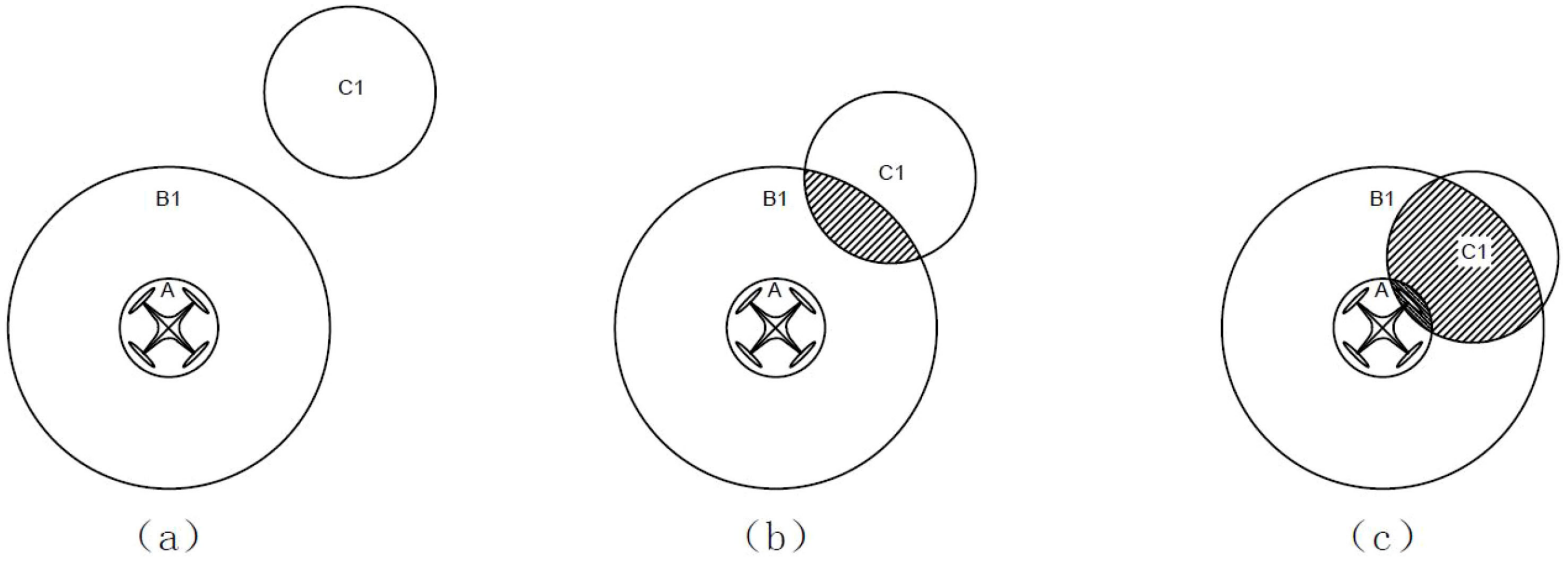
Topological situations between the UAV, detection region, and obstacles: **(A)** No intersection among the three; **(B)** Obstacle within the detection region; **(C)** Obstacle intersects with the UAV.

According to the R5DOS model, this study provides the following definitions. For the detection region B, it must contain the UAV body A, satisfying the PP topological relation, denoted as PP(B, A). As for the relationship between obstacles, the UAV, and the detection region, three possible scenarios are most likely to occur, as illustrated in **Figure 2**.

In the figure, A represents the UAV body region, B_1_ represents the detection region, and C_1_ represents the obstacle. **Figure 2A** illustrates the scenario where the obstacle does not intersect with the detection region or the UAV, indicating that the UAV has not detected any obstacles and is in a relatively safe state. The corresponding topological relation matrix is denoted as $R5_{a}=\left(\begin{array}{cccc} 0 & 1 & 0 & 0\\ 0 & 1 & 1 & 1 \end{array}\right)$ . **Figure 2B** depicts the scenario where the obstacle is within the detection region but has not yet intersected with the UAV. In this case, the obstacle is inside the detection region but has not collided with the UAV, placing the UAV in a relatively dangerous situation. The corresponding topological relation matrix is denoted as $R5_{b}=\left(\begin{array}{cccc} 0 & 1 & 0 & 0\\ 1 & 1 & 1 & 1 \end{array}\right)$ . **Figure 2C** illustrates the scenario where the obstacle is within the detection region and intersects with the UAV. In this case, the UAV collides with the obstacle, posing a dangerous situation. The corresponding topological relation matrix is denoted as $R5_{c}=\left(\begin{array}{cccc} 1 & 1 & 0 & 0\\ 1 & 1 & 1 & 1 \end{array}\right)$ .

### 2.2 Modification of the R5DOS spatial topological relationship model

To better represent the spatial relationships between UAVs and obstacles, an improvement to the R5DOS model is essential. The induction matrix of the R5DOS model is divided into the R5 layer and the DOS layer, which are used to express topological and spatial relationships, respectively. For detected obstacles, the UAV needs to store their information to determine the location of the obstacles and their corresponding topological spatial relationships. However, the R5DOS model can only express the topological relationships of three simple objects, which is evidently impractical for high-density obstacle maps. To better express and store the topological spatial relationships between multiple obstacles and UAVs, we have modified the DOS layer of the R5DOS model to be a 4×4 matrix, defined as follows.


(1) $$DOS=\left(\begin{array}{cccc} 1NE & 2NE & 3NW & 4NW\\ 1EN & 2EN & 3NW & 4NW\\ 5ES & 6ES & 7WS & 8WS\\ 5SE & 6SE & 7SW & 8SW \end{array}\right)$$

The spatial relationships corresponding to each matrix element are as follows.


(2) $$\begin{array}{l} \{\begin{array}{c} 1NE;x_{c}\geq 0,y_{c}\geq 0,z_{c}\geq 0,\theta_{ac}\in \left[0,\frac{\pi }{4}\right)\\ 2NE;x_{c}<0,y_{c}\geq 0,z_{c}\geq 0,\theta_{ac}\in \left[0,\frac{\pi }{4}\right)\\ 1EN;x_{c}\geq 0,y_{c}\geq 0,z_{c}\geq 0,\theta_{ac}\in \left[\frac{\pi }{4},\frac{\pi }{2}\right)\\ 2EN;x_{c}<0,y_{c}\geq 0,z_{c}\geq 0,\theta_{ac}\in \left[\frac{\pi }{4},\frac{\pi }{2}\right) \end{array}\;\{\begin{array}{c} 5ES;x_{c}\geq 0,y_{c}\geq 0,z_{c}<0,\theta_{ac}\in \left[\frac{\pi }{2},\frac{3\pi }{4}\right)\\ 6ES;x_{c}<0,y_{c}\geq 0,z_{c}<0,\theta_{ac}\in \left[\frac{\pi }{2},\frac{3\pi }{4}\right)\\ 5SE;x_{c}\geq 0,y_{c}\geq 0,z_{c}<0,\theta_{ac}\in \left[\frac{3\pi }{4},\pi \right)\\ 6SE;x_{c}<0,y_{c}\geq 0,z_{c}<0,\theta_{ac}\in \left[\frac{3\pi }{4},\pi \right) \end{array}\\ \{\begin{array}{c} 8SW;x_{c}\geq 0,y_{c}\geq 0,z_{c}<0,\theta_{ac}\in \left[\pi ,\frac{5\pi }{4}\right)\\ 7SW;x_{c}\geq 0,y_{c}\geq 0,z_{c}\geq 0,\theta_{ac}\in \left[\pi ,\frac{5\pi }{4}\right)\\ 8WS;x_{c}\geq 0,y_{c}<0,z_{c}<0,\theta_{ac}\in \left[\frac{5\pi }{4},\frac{3\pi }{2}\right)\\ 7WS;x_{c}<0,y_{c}<0,z_{c}<0,\theta_{ac}\in \left[\frac{5\pi }{4},\frac{3\pi }{2}\right) \end{array}\;\{\begin{array}{c} 4WN;x_{c}\geq 0,y_{c}<0,z_{c}\geq 0,\theta_{ac}\in \left[\frac{3\pi }{2},\frac{7\pi }{4}\right)\\ 3WN;x_{c}<0,y_{c}<0,z_{c}\geq 0,\theta_{ac}\in \left[\frac{3\pi }{2},\frac{7\pi }{4}\right)\\ 4NW;x_{c}\geq 0,y_{c}<0,z_{c}\geq 0,\theta_{ac}\in \left[\frac{7\pi }{4},2\pi \right)\\ 3NW;x_{c}<0,y_{c}<0,z_{c}\geq 0,\theta_{ac}\in \left[\frac{7\pi }{4},2\pi \right) \end{array} \end{array}$$

Wherein, 1-8 represent the eight hexagram limits in three-dimensional space, and “c” represents an obstacle. $\theta_{ac}$ represents the dihedral angle between the UAV (a) and the obstacle (c). Therefore, regardless of the number of obstacles, as long as an obstacle appears in any octant, it can be recorded in the matrix. Thus, irrespective of how many obstacles are present in a particular octant, we can provide the following definitions.


(3) $$\epsilon (DOS)=\{\begin{array}{c} 0:\text{ There are no obstacles in the area}\\ n:\text{ There are n obstacles in the area} \end{array}\;n\in [1,+\infty )$$

Through the improvements made to the R5DOS model, we gain the capability to articulate the spatial relationships between UAV and any number of obstacles. Consequently, the refined R5DOS model allows for a comprehensive representation of the spatial topology including UAVs, obstacles, and target points. Building upon the improvements introduced in the D5DOS model, we can further refine the node selection process of the A-Star algorithm.

### 2.3 Introduction to A-star algorithm and corresponding improvements

The space complexity of the traditional A-star exhibits exponential growth, showing a noticeable increase in computational requirements as the map size expands (Li et al., 2022). This issue primarily manifests during the execution of the A-star algorithm, where a substantial number of nodes are visited without considering whether these nodes are relevant to the final path. Consequently, this further increases the algorithm’s runtime. To address this challenge, this study introduces a custom feature attention mechanism to enhance the A-star algorithm. In the A-star algorithm, its cost function, typically denoted as ‘ $F_{cot}\left(n\right)$ ’, is commonly expressed through the expression ‘ $F_{cot}\left(n\right)=H\left(n\right)+G\left(n\right)$ ’. In this study, we employed Euclidean distance to calculate the movement cost, which is particularly suitable for the representation of three-dimensional space using the grid-based approach. Here, ‘ H(n) ’ represents the cost function for estimating the path from the nth feature point to the target, while ‘ G(n) ’ denotes the movement cost function for the shortest path from the starting point to the nth feature point. For the grid-based representation of three-dimensional space, the formula for calculating the movement cost ‘ f(n) ’ is given by Equation 4.


(4) $$f(n)=\sqrt{{\left(x_{n}-x_{n-1}\right)}^{2}+{\left(y_{n}-y_{n-1}\right)}^{2}+{\left(z_{n}-z_{n-1}\right)}^{2}}$$

**Figure 3:**
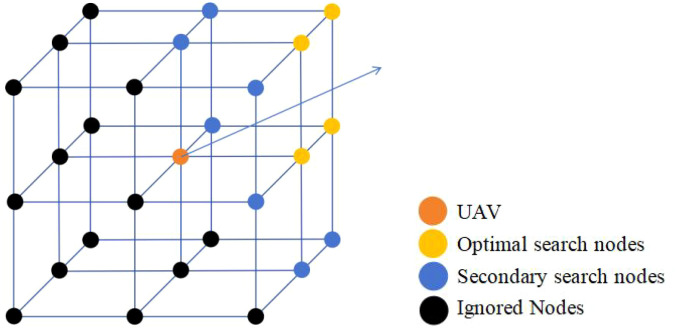
Search mechanism of the improved A-star algorithm.

Here, ‘ $\left(x_{n},y_{n},z_{n}\right)$ ’ and ‘ $\left(x_{n-1},y_{n-1},z_{n-1}\right)$ ’ represent the coordinates of nodes ‘n’ and ‘n-1’, respectively. This study redefines the nodes for UAV path search. In three-dimensional space, the number of nodes that a UAV can choose for its next move is significantly greater than the cardinal directions available in two-dimensional space. To better apply the A-star algorithm, this study redivides the space into 16 regions based on the R5DOS model. However, this partitioning method is not very friendly to the node evaluation of the A-star algorithm. To improve this, this study is based on eight octants, assuming the orange point represents the UAV’s position. In this case, all vertices of the neighboring octants can be considered as neighboring nodes for the current node, resulting in a total of 26 potential search nodes. Having too many nodes can impact the efficiency of the algorithm. Therefore, based on the UAV’s next move direction, the study filters out the closest four nodes (yellow points). After the first round of searching does not yield the optimal node, it then searches for the neighbors of the yellow nodes (blue points), as illustrated in **Figure 3**.

Simultaneously, based on the definition of the improved DOS layer, we can further enhance the search mechanism. By retrieving the spatial topology matrix of the current UAV, the R5 layer is divided into two scenarios.

In case $R5=\left(\begin{array}{cccc} 0 & 1 & 0 & 0\\ 0 & 1 & 1 & 1 \end{array}\right)$ is satisfied, employ the search method illustrated in **Figure 3** to filter nodes.

In case $R5=\left(\begin{array}{cccc} 0 & 1 & 0 & 0\\ 1 & 1 & 1 & 1 \end{array}\right)$ is satisfied, indicating the detection of an obstacle, under the search mechanism depicted in **Figure 3**, proceed to eliminate nodes with non-zero DOS. By redefining the method for searching nodes, it is possible to significantly reduce unnecessary node searches, thereby enhancing the search efficiency of the improved algorithm in three-dimensional environments. This approach facilitates the more effective identification of the optimal path.

### 2.4 Feature attention mechanism

**Figure 4:**
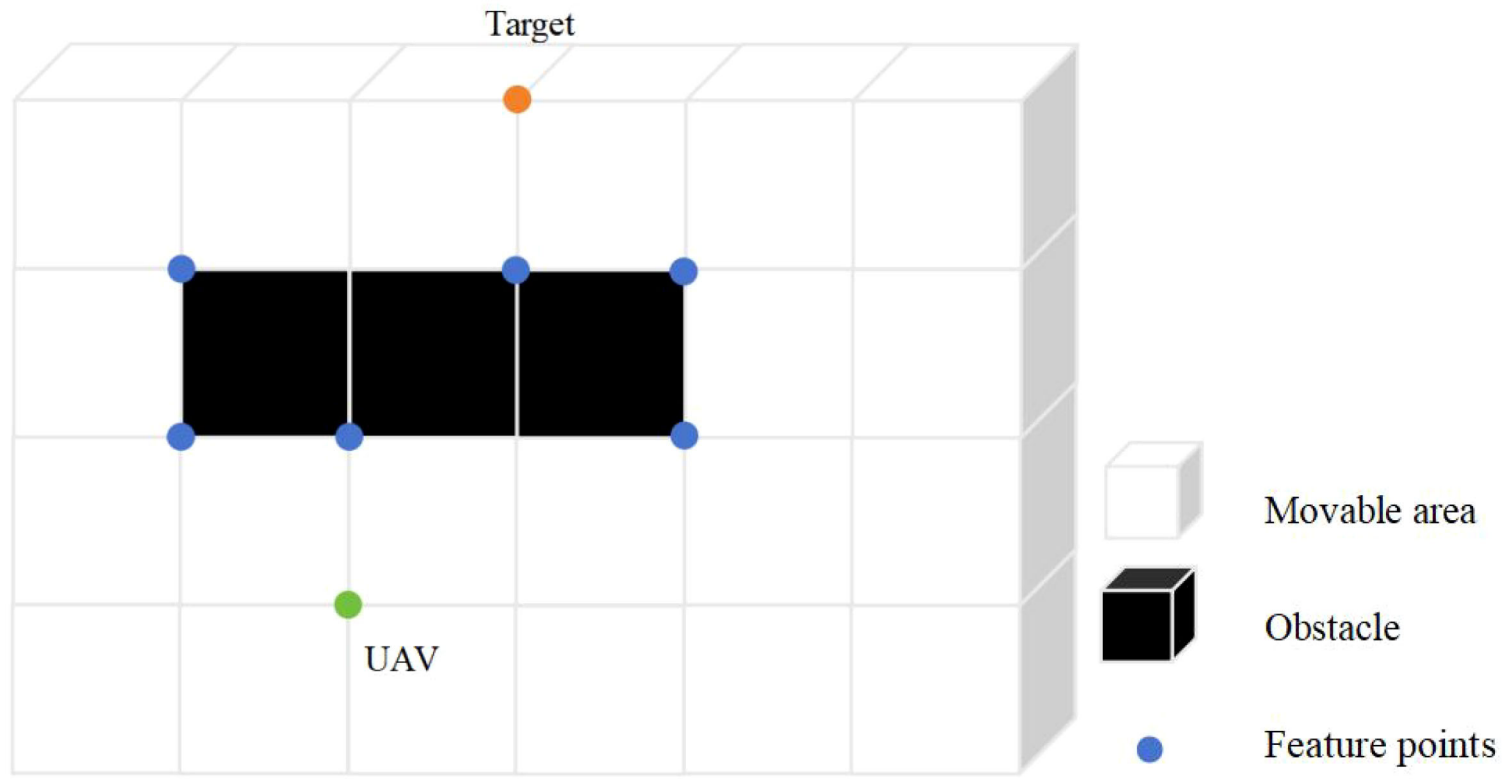
The custom feature attention mechanism governs the process of selecting feature points.

Although the R5DOS model-based search mechanism defined in Section 2.2 can eliminate most unnecessary nodes, there are still a significant number of invalid nodes in three-dimensional space that cannot be filtered out using this method. Therefore, this study incorporates a feature attention mechanism module into the A-Star algorithm, which is activated when the UAV detects obstacles. After integrating this module, the UAV focuses more on the local features of obstacles during path planning. The dual-node filtering algorithm, combining the R5DOS model with the feature attention mechanism, effectively removes a large number of unnecessary nodes, as shown in **Figure 4**.

In the illustration, green nodes represent the UAV $\left(x_{UAV},y_{UAV},z_{UAV}\right)$ , orange nodes represent the target point $\left(x_{T},y_{T},z_{T}\right)$ and black blocks represent obstacles. When a UAV needs to avoid obstacles in a three-dimensional space, it must focus on the primary characteristics of the obstacles, particularly the vertices and special points along the edges. By concentrating on these features and ignoring other insignificant nodes, the UAV can significantly reduce its computational load, thus alleviating much of the processing burden. For obstacles in three-dimensional space, their features include vertices and edges. Therefore, based on these features, the following definitions are made. The obstacle grid representation is denoted as Gobs(n) $\left(x_{G},y_{G},z_{G}\right)$ , with a value of 1 for obstacle grids, and the non-obstacle grid representation is denoted as GN-obs(n), with a value of 0. This study defines a feature attention estimation function, A(n), which represents the number of adjacent obstacles to the nth node. Typically, a node has 8 adjacent grids, and A(n) is expressed as formula 5.


(5) $$A(n)=\sum G_{obs(n)},A(n)\subset [0,8]$$

For feature points, the following definitions can be obtained:

The evaluation function for the characteristic points at the vertices is: A(n)=1 .
2.The evaluation function for the characteristic points located along the edges of the obstacles is defined as: $A(n)\in [2,7]\cap \min[f_{G-U}(n)]$ or $A(n)\in [2,7]\cap \min[f_{T-G}(n)]$ .

‘ $f_{G-U}(n)$ ’ represents the path cost function between obstacles and the UAV, while ‘ $f_{T-G}(n)$ ’ denotes the path cost function between the target point and obstacles. According to the above definitions, the feature points that can be filtered by this attention mechanism include the vertices of obstacles and the points on the edges of obstacles that are closest to the UAV or the target point. The characteristic points that meet the conditions are stored in List $A_{l}(n)$ . By employing this filtering method, the UAV can focus on the critical features of the obstacles that need to be avoided, thereby reducing the interference of irrelevant nodes in the algorithm. During path planning, the characteristic points in List $A_{l}(n)$ are prioritized for visitation. First, the R5DOS model filters out most of the nodes, followed by the feature attention mechanism, which further filters the nodes around the obstacles. This approach allows the UAV to focus on the key features of obstacles that need to be avoided, thereby reducing the interference of irrelevant nodes in the algorithm.

Finally, we conduct a two-sample t-test on the algorithm results to analyze the simulated experimental outcomes, considering whether the differences between the proposed algorithm and other algorithms are significant. The formula for the t-test statistic for independent samples is shown below:


(6) $$t=\frac{{\bar{X}}_{1}-{\bar{X}}_{2}}{\sqrt{\frac{(n_{1}-1)S_{1}^{2}+(n_{2}-1)S_{2}^{2}}{n_{1}+n_{2}-2}(\frac{1}{n_{1}}+\frac{1}{n_{2}})}}$$

In which, $S_{1}^{2}$ and $S_{2}^{2}$ represent the sample variances of two sets of data, while $n_{1}$ and $n_{2}$ denote the sample sizes.

### 2.5 RFA-star algorithm

Building on the preceding definitions, this paper provides the definition of the RFA-Star algorithm. Detection is performed within the exploration area, and the environmental and obstacle detection results are stored in the feature matrix of R5DOS. If the UAV detects an obstacle, a custom feature attention mechanism is introduced to search for the feature points of the obstacle. This scenario is defined as condition A.


(7) Condition A: ∑ϵ(DOS)≥4

‘ ϵ(DOS) ’ represents the DOS layer of the R5DOS model (Li et al., 2023). When the UAV encounters conditions corresponding to Condition A, the local map of the detection area is gridded. An improved A-star algorithm and a feature attention module are then utilized to filter feature points. Subsequently, guiding the UAV through obstacle avoidance involves a process outlined by the RFA-Star algorithm, as illustrated in the following steps.

1. Initialize the map.
2. Detect the topological relationships of the R5 layer to determine the presence of obstacles. If obstacles are detected, check if condition A is satisfied.
3. If Condition A is met, a feature attention mechanism is introduced to visit the characteristic points in List $A_{l}(n)$ .
4. Repeat steps 2-4 until the UAV has searched all characteristic points or has escaped from the minimum value trap.
5. Calculate the cost function for all nodes and select the optimal path.
6. The UAV moves according to the nodes until it reaches the target location.

Based on the above process, the corresponding pseudocode of the algorithm is provided in **Table 1**.

**Table 1:** Pseudocode of the Algorithm.

RFA-Star Algorithm Pseudocode
1 initialize_map()
2 while not search_complete:
3 topological_relationships = detect_topological_relationships(R5_layer)
4 if obstacles_present(topological_relationships):
5 if satisfies_condition_A(topological_relationships):
6 continue
7 else:
8 feature_points = feature_attention_module()
9 update_search_status()
10 calculate_cost_function()
11 optimal_path = select_optimal_path()
12 for node in optimal_path:
13 move_UAV_to(node)
14 end if
15 end if
16 end while

**Figure 5:**
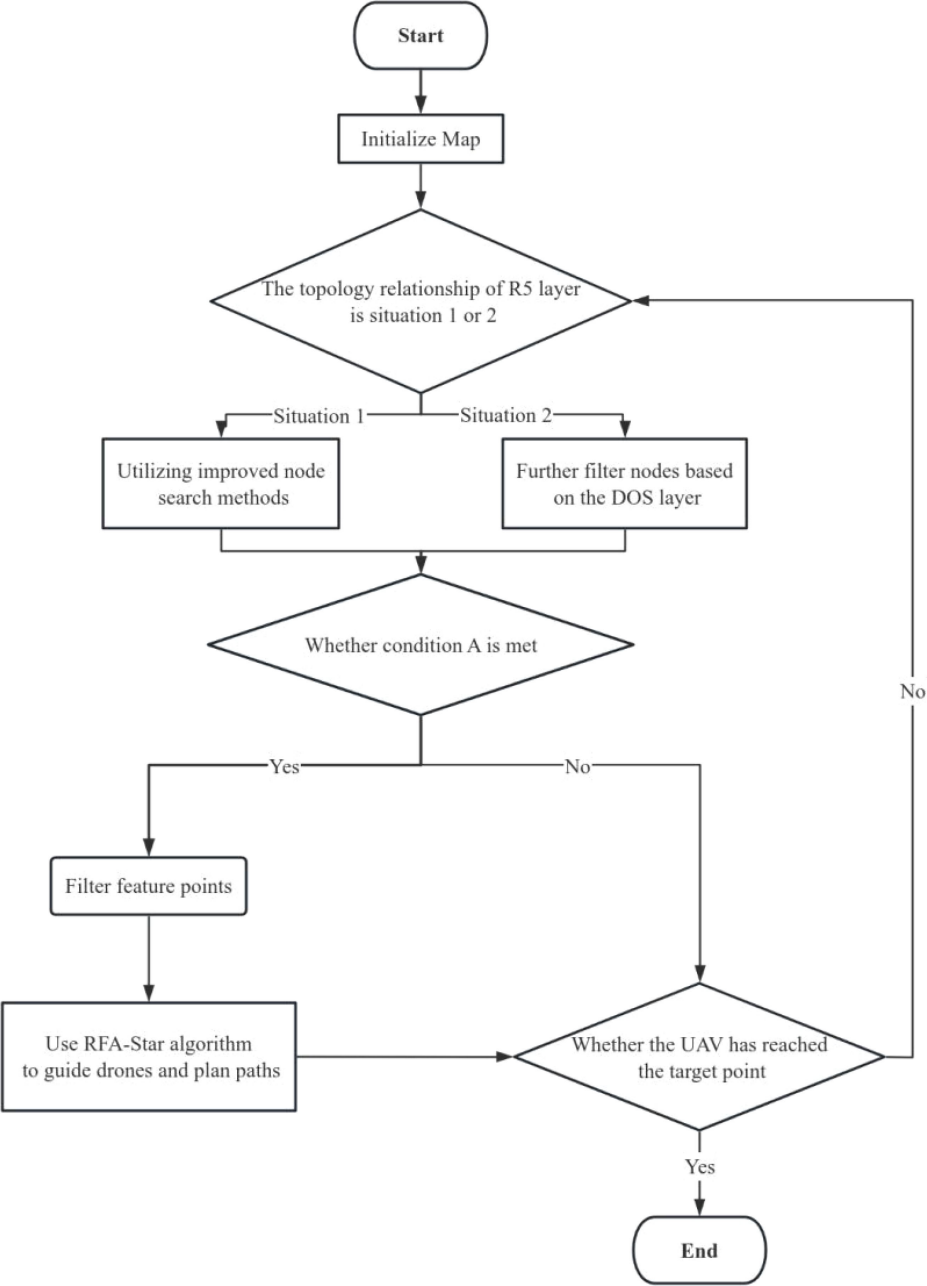
Flowchart of PARA-Star algorithm.

**Figure 5** illustrates the flowchart of the algorithm.

## 3 Results

### 3.1 Experimental details

**Figure 6:**
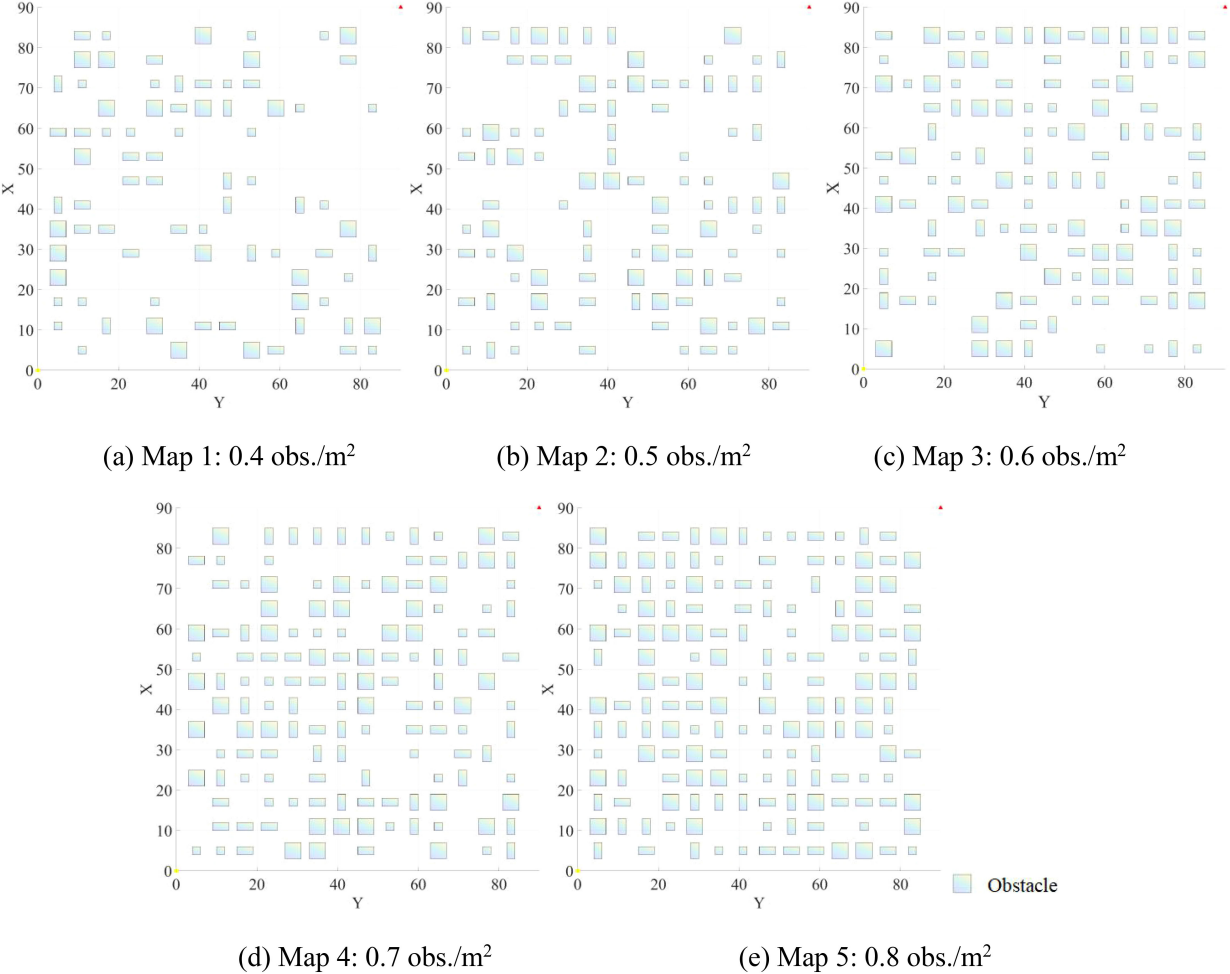
Projections of maps on the xy-plane at varying obstacle densities: **(A)** 0.4 obs./m^²^; **(B)** 0.5 obs./m^²^; **(C)** 0.6 obs./m^²^; **(D)** 0.7 obs./m^²^; **(E)** 0.8 obs./m^²^.

To assess the efficacy of the RFA-Star algorithm in path planning within high-density obstacle environments and to compare its performance with other algorithms, this study designed two experiments:

1. In a randomly generated map with dimensions of 90m × 90m × 15m, five different obstacle densities were deployed, ranging from 0.4 to 0.8 obstacles per square meter (obs./m²). **Figures 6 (A–E)** illustrates the projections of these maps on the xy-plane at varying obstacle densities.
2. For the second experiment, five maps with varying lengths and widths but the same height were created, all with a fixed obstacle density of 0.8 obs./m². The specific details of these maps are provided in **Table 2**.

**Table 2:** Detailed settings information for different maps.

Map name	Map size	obstacle density(obs./m^2^)	Target point
Map 1	50m×50m×15m	0.8	(50,50,15)
Map 2	60m×60m×15m	0.8	(60,60,15)
Map 3	70m×70m×15m	0.8	(70,70,15)
Map 4	80m×80m×15m	0.8	(80,80,15)
Map 5	90m×90m×15m	0.8	(90,90,15)

This study undertook a comparative analysis of the RFA-Star algorithm against other state-of-the-art spatial reasoning-based path planning methods, namely RJA-Star (Li et al., 2023) Improved A-Star (Li et al., 2022)., and the A-Star. The RJA-Star integrates an enhanced jump point search algorithm into the A-Star, effectively diminishing the number of nodes, computation time, and computational complexity. In contrast, the Improved A-Star algorithm enhances the A-Star by incorporating the R5DOS model to reduce search nodes. Furthermore, given the similar path-searching approaches employed by the RFA-Star and RJA-Star algorithms, this study extends the comparison to include A-Star, RJA-Star, and RFA-Star across five distinct map sizes. Detailed map information is available in **Table 2**.

For better comparison, this study set the obstacle density of the map to 0.8 obs./m^2^, with the starting point designated as (0, 0, 0). MATLAB was employed in this research to randomly generate obstacle maps, and all simulations were conducted on a 13th Gen Intel(R) Core(TM) i5-13600KF 3.50 GHz CPU and NVIDIA GeForce RTX 4080 GPU.

### 3.2 Results Comparison for Different Obstacle Densities

For each obstacle density level, this simulation experiment generated 10 distinct maps randomly, with the recording of three experimental outcomes: average flight distance, computation time, and the number of search nodes. To maintain variable consistency, this study set the starting coordinates at (0,0,0) and the target coordinates at (90,90,15).

**Figure 7:**
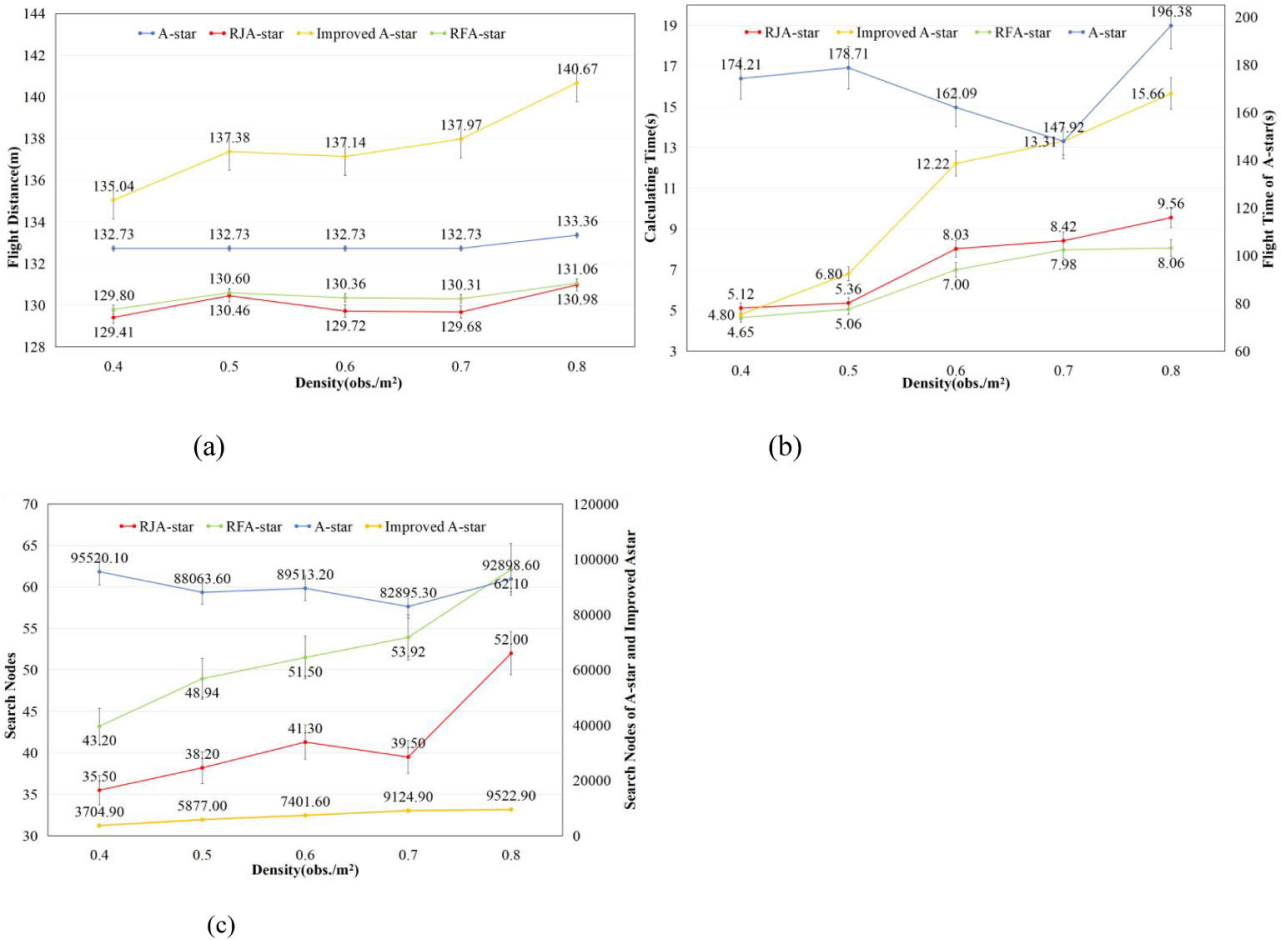
Results of four path planning algorithms in maps generated with different obstacle densities.

As illustrated in **Figure 7**, the RFA-Star algorithm’s flight distance is only 0.06% to 0.49% longer than that of the RJA-Star algorithm, but 3.88% to 6.83% shorter than that of the Improved A-Star algorithm. The number of explored nodes is 19.42% to 36.51% higher than that of RJA-Star, while it accounts for only 0.59% to 1.17% of the nodes explored by Improved A-Star. The computation time for RFA-Star ranges from 84% to 94% of that required by RJA-Star, and from 51% to 96% of that required by Improved A-Star. While RFA-Star shows minimal differences from RJA-Star in terms of flight distance and explored nodes, it significantly outperforms RJA-Star in computation time. For UAVs, the ability to quickly and efficiently generate safe paths is crucial. Compared to the other three path planning algorithms, RFA-Star demonstrates the capability to swiftly generate high-quality paths while maintaining relatively shorter routes.

**Figure 8:**
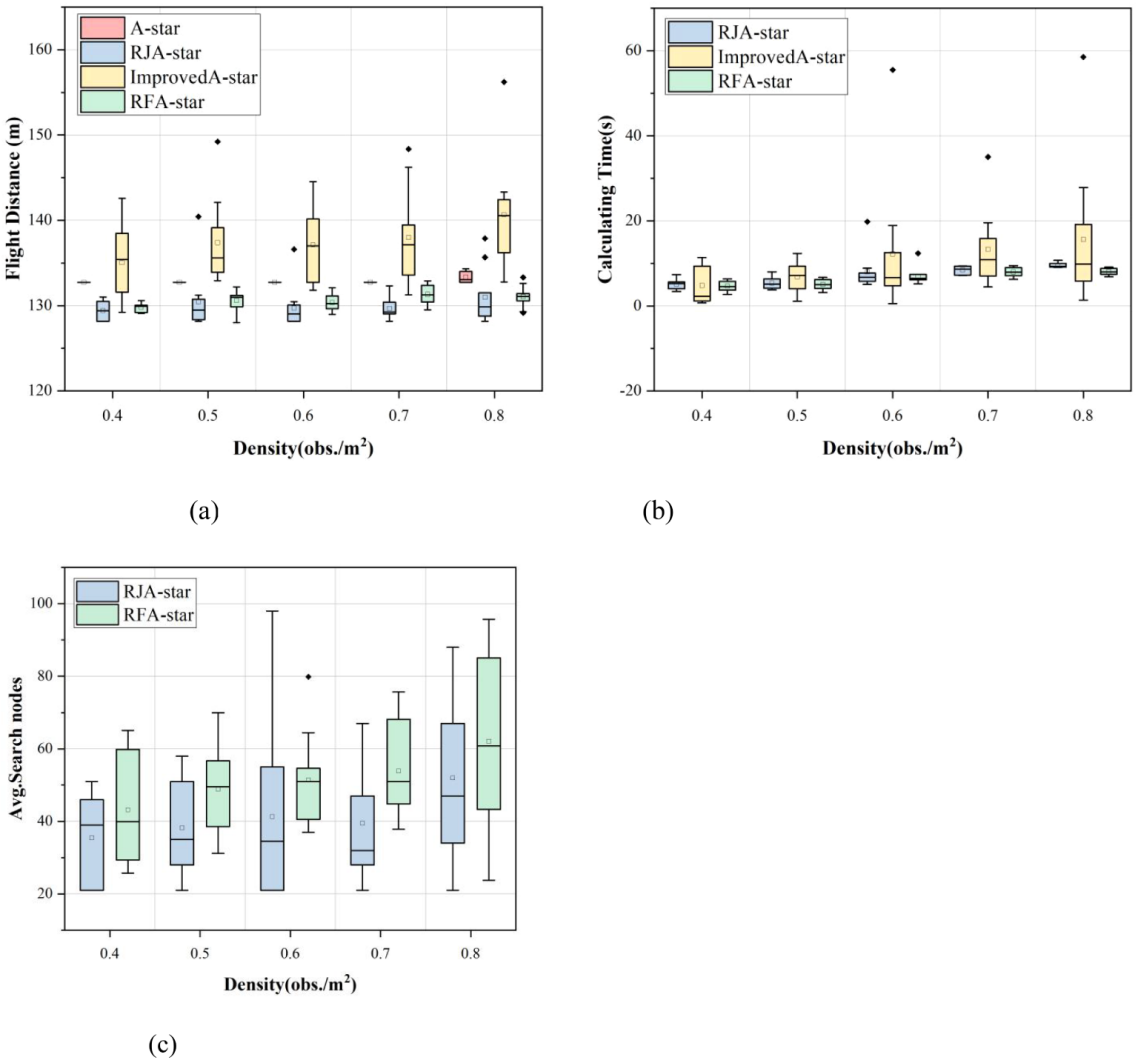
Comparison of the distribution of experimental results of four path planning algorithms under different obstacle densities.

This study presents distribution plots of the experimental results for flight distance, computation time, and search nodes. It is important to note that, due to the significantly higher computation time and search nodes associated with the A-Star and Improved A-Star algorithms compared to the RJA-Star and RFA-Star algorithms, corresponding distribution plots for A-Star and Improved A-Star were not generated for clarity, as shown in **Figure 8**. The box plots of the experimental results reveal that the RFA-Star algorithm demonstrates more concentrated outcomes across various obstacle densities compared to the other three path planning algorithms, with less fluctuation in results due to changes in the environment. However, as observed in **Figure 8A**, the A-Star algorithm maintains a nearly consistent flight distance across maps of the same size. Although it does not achieve the shortest distances in this study, its stability is one of the reasons why the A-Star algorithm has become a classic.

We conducted a t-test to analyze the search node results of the RJA-Star algorithm and the RFA-Star algorithm, with the findings presented in **Table 3**. The t-test was performed with a significance level of 0.05 to determine if there were statistically significant differences in the number of search nodes between the two algorithms across various obstacle densities.

**Table 3:** T-test results of the RJA-Star algorithm and the RFA-Star algorithm.

Density(obs./m^2^)	H	P-value
0.4	0	0.22
0.5	0	0.09
0.6	0	0.25
0.7	1	0.04
0.8	0	0.35

As shown in **Table 3**, at obstacle densities of 0.4, 0.5, 0.6, and 0.8, no significant difference was observed (H = 0), indicating that the two algorithms perform similarly in these conditions. However, at an obstacle density of 0.7, a significant difference was detected (H = 1, p = 0.04), suggesting that the algorithms differ in their efficiency or behavior under this specific condition. The t-test thus highlights where the algorithms diverge in performance, particularly in their handling of search nodes.

### 3.3 Results Comparison for Different Map Sizes

**Figure 9:**
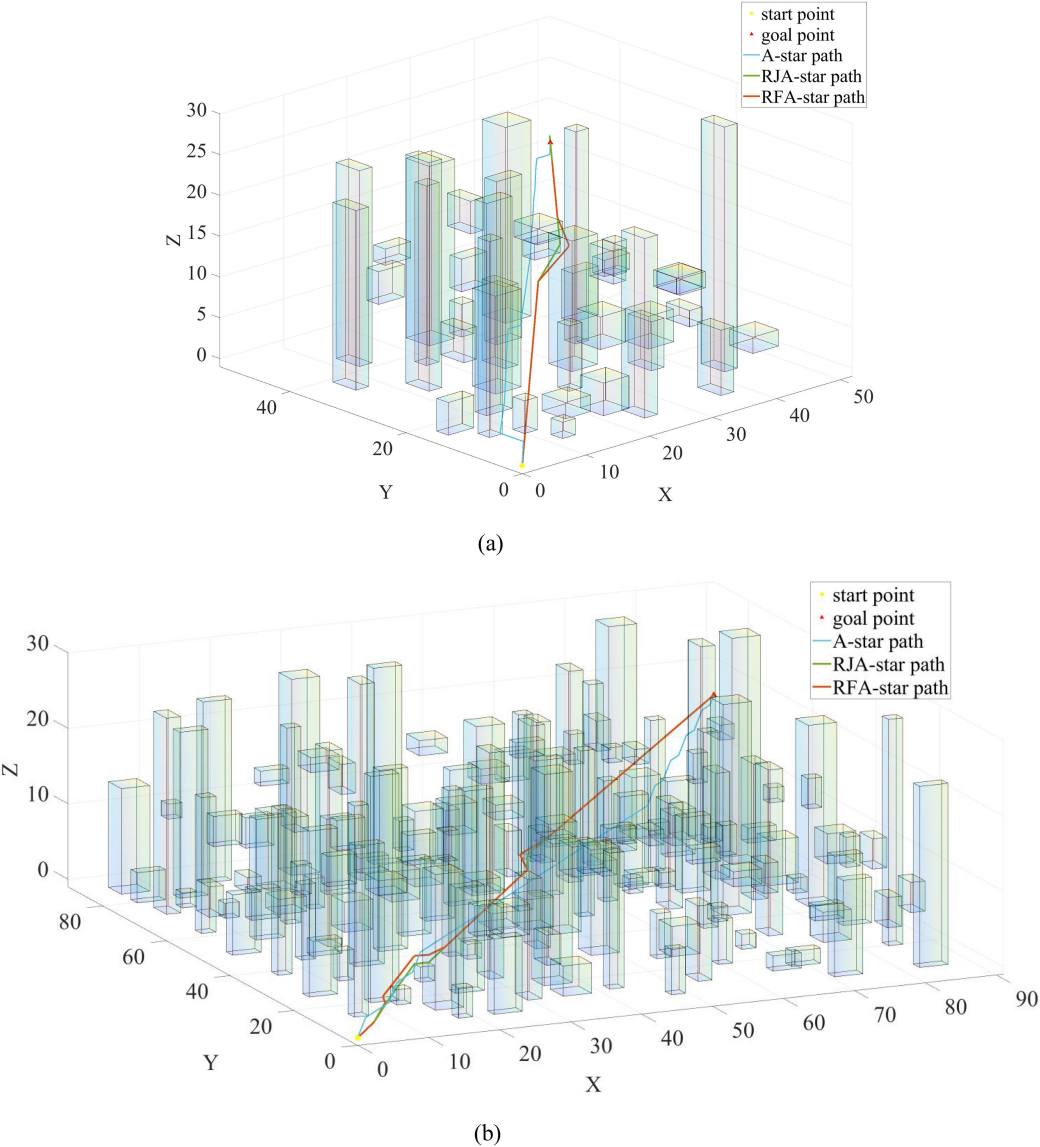
Trajectories of A-Star (blue), RJA-Star (red) and RFA-Star (green) under different map sizes.

Since the RFA-Star, RJA-Star, and A-Star can all utilize grid maps for search, this study evaluated the effectiveness of these three algorithms under different map sizes. Under the precondition that the obstacle density across all maps is set to 0.8 obs./m², the paper compares the flight distance, computation time, and search nodes of these three algorithms across five different map sizes. Ten maps were randomly generated for each size to conduct experimental comparisons. As depicted in **Figure 9**, the path planning scenarios for Map 1 and Map 5 are illustrated.

In **Figure 9**, it is shown that among the results of Map1 and Map5, the A-star algorithm produces the longest path compared to the other three algorithms. Additionally, as depicted in **Figure 9B**, the A-star algorithm generates a wavy path for obstacle avoidance, which is not in line with typical UAV motion patterns. Although the RJA-star algorithm results in the shortest path, it does so by closely hugging obstacles during avoidance. This approach compromises the UAV’s safety in favor of a shorter route. However, in practical UAV operations, this close proximity to obstacles is highly dangerous, increasing the risk of collision. Therefore, considering both safety and flight distance, the RFA-star algorithm offers the most balanced and optimal solution by planning a relatively short path while ensuring safety.

From the t-test results and the findings presented in **Table 4**, it is evident that influenced by the map size, the values of all three experimental metrics exhibit an increasing trend. Particularly noteworthy is the exponential increase observed in the average computation time and average search nodes for the A-Star algorithm. From the results, it can be observed that the average flight distance of the RFA-Star algorithm proposed in this study is comparable to that of the RJA-Star algorithm. Although the average search nodes for RFA-Star are higher than those for RJA-Star, it outperforms the other two algorithms in the crucial aspect of computation time. Moreover, in different map sizes, the average computation time of the RFA-Star algorithm is 85% to 92% of that of the RJA-Star algorithm, indicating a significant improvement. This suggests that the RFA-Star algorithm, even with an increase in map size, can maintain stable computation performance, providing rapid and stable path planning.

**Table 4:** Comparison of three path planning methods.

	Path Planning Algorithm	Avg.Flight Distance(m)	Avg.Calculating Time(s)	Avg.Search nodes
Map 1	A-star	76.16	24.86	30746
	RJA-star	73.16	4.55	20
	RFA-star	73.28	4.06	24
Map 2	A-star	90.46	48.47	44682
	RJA-star	87.20	5.70	30
	RFA-star	87.32	5.09	35
Map 3	A-star	104.52	70.12	55454
	RJA-star	101.05	6.67	32
	RFA-star	102.13	5.93	38
Map 4	A-star	118.66	95.98	64092
	RJA-star	117.08	7.67	48
	RFA-star	118.61	6.35	56
Map 5	A-star	132.81	143.72	80401
	RJA-star	130.92	9.36	51
	RFA-star	131.97	9.17	59

## 4 Discussion

This paper establishes a UAV path planning model based on spatial topological relationships, offering rapid and stable path planning services for UAVs operating in high-density obstacle environments. Leveraging the R5DOS model, the study improves the A-Star algorithm by introducing a feature attention mechanism to enhance obstacle avoidance capabilities during UAV flight operations. In the path planning process, the map is first initialized. Subsequently, topological relationships are detected in the R5 layer to identify obstacle presence. If obstacles are present, condition A is checked to determine the need for obstacle avoidance. If condition A is satisfied, the feature attention mechanism is introduced, and path planning is conducted by selecting characteristic points. The process continues until the UAV completes the search for all characteristic points. Next, by calculating the cost function for all nodes and considering both the cost of the path and the importance of characteristic points, the path with the minimum cost is chosen as the optimal path. Finally, the UAV moves along the nodes of the optimal path until reaching the destination point. The comprehensive application of topological relationship detection, feature attention mechanism, and A-Star in the RFA-Star algorithm achieves efficient and intelligent UAV path planning. Additionally, it enables the rapid and stable planning of UAV operational paths.

To further validate the proposed path planning algorithm’s capability to achieve rapid and stable target reachability in complex, high-density obstacle environments, this study conducted a series of comparative experiments. The RFA-Star algorithm was compared against the A-Star algorithm and two other state-of-the-art spatial reasoning-based path planning algorithms under five different obstacle density conditions. From the experimental results we can draw the following conclusions: (1) the RFA-Star boasts the shortest computation time, approximately 84%-94% less than the RJA-Star and 51%-96% less than the Improved A-Star. The flight distance is comparable to that of the RJA-Star algorithm, with only a slight difference, while the search nodes are slightly higher than those of the RJA-Star algorithm. However, these three results are significantly lower than those obtained with the Improved A-Star and the A-Star. (2) The RJA-Star has the fewest searched nodes, as it selects only the nodes closest to the line connecting the UAV and the target during the node search. However, this approach still has the potential to fall into the minimum value trap because it places greater emphasis on computing the nearest nodes. (3) The experimental results for the Improved A-Star show a dispersed distribution, indicating that the algorithm is not stable. This instability becomes more pronounced with increasing obstacle density. Although the number of visited nodes is lower than that of the A-Star, it is still significantly higher than that of the RFA-Star and the RJA-Star. (4) The A-Star, being a traditional and classic algorithm, exhibits not the shortest but the most stable flight distance among the four algorithms. In the first 40 random experiments, the flight distance presented by the A-Star remains constant. Only in the random map with an obstacle density of 0.8 obs./m^2^ did different results emerge, with a highly concentrated distribution of flight distances. However, due to its limitations, the computation time and search nodes of the A-Star algorithm are significantly greater than those of the other three algorithms.

The experimental results demonstrate that the RFA-Star algorithm exhibits strong robustness under varying obstacle densities. Across three key metrics—flight distance, computation time, and the number of search nodes—the RFA-Star algorithm shows relatively stable performance, with minimal impact from environmental changes. Compared to other algorithms, RFA-Star’s results fluctuate less, particularly in high obstacle density scenarios (e.g., 0.8 obs./m²), where both flight distance and computation time remain within reasonable limits, indicating good stability. Experiments conducted on maps of different sizes further validate the robustness of the RFA-Star algorithm. Although all algorithms show an upward trend in average flight distance, computation time, and the number of search nodes as map size increases, RFA-Star maintains a significant advantage in computation time. Even with larger map sizes (as shown in **Figure 9B**), RFA-Star’s computation time is only 85% to 92% of that of the RJA-Star algorithm. This indicates that the RFA-Star algorithm can maintain stable computational performance as map size increases, ensuring fast and stable path planning. Additionally, the experimental results show that although the average number of search nodes in RFA-Star is slightly higher than in the RJA-Star algorithm, its overall performance still surpasses other algorithms, especially in terms of the critical metric of computation time. This stability and efficiency across various map sizes further confirm the robustness of the RFA-Star algorithm in diverse and complex scenarios.

In summary, the RFA-Star algorithm demonstrates a relatively short computation time, comparable flight distance to the RJA-Star algorithm, with slightly more search nodes. The RFA-Star algorithm exhibits good performance in terms of computational efficiency and the balance between path quality, but further optimization is needed to overcome its drawbacks.

### 4.1 Comparison with Other Path Planning Algorithms

To further validate the effectiveness and advancement of the RFA-Star algorithm, we conducted a series of comparative experiments with other state-of-the-art path planning algorithms. Although the unique nature of RFA-Star made it challenging to find directly comparable algorithms, this approach allowed us to place RFA-Star within the broader context of modern path planning techniques. By comparing RFA-Star with diverse algorithms designed for different environments and operational requirements, we could better understand its strengths and limitations. This comparison not only highlights the robustness and efficiency of RFA-Star in various scenarios but also provides a comprehensive perspective on its relative performance against other leading methods.

Castro et al. (Castro et al., 2023) combined Rapidly-exploring Random Trees (RRT) with Deep Reinforcement Learning (DRL) to generate and control UAV trajectories during the inspection of olive fly traps. Their proposed solution was tested in a simulated environment with 10 dynamic obstacles within a 300 cubic meter area. The RRT+DQN algorithm achieved an average runtime of 8.2 milliseconds, outperforming traditional algorithms like Genetic Algorithm (GA) and Dijkstra, which had runtimes of 8.7 milliseconds and 2.4 milliseconds, respectively. The pure RRT algorithm had a runtime of 6.5 milliseconds. Souto et al. (Souto et al., 2023) developed a novel reinforcement learning-based method aimed at reducing power consumption during UAV missions in disaster scenarios to mitigate the negative effects of changing wind directions. Compared to simpler heuristic methods, the power-saving effect was reduced by 15.93%. The study showed that Q-learning using an ϵ-greedy decay method was the most efficient, resulting in shorter mission durations compared to SARSA and basic Q-learning. While the main focus of this study was on energy efficiency rather than path planning speed, it highlighted the importance of algorithm efficiency in extending UAV mission life. Xu et al. (Xu et al., 2024) proposed a bionic 3D path planning algorithm for agricultural UAVs, designed to optimize safe flight paths between work plots obstructed by multiple obstacle zones. The algorithm was tested in a 100 cubic meter irregular hilly space with several randomly placed obstacles. The experimental results showed that the bionic 3D path planning reduced path length by 75.15%, and energy consumption decreased by 13.91% to 27.35% compared to other algorithms, including Ant Colony Optimization and Artificial Bee Colony algorithms. The specific results are presented in **Table 5**.

**Table 5:** Comparative Analysis of Path Planning Algorithms for UAVs in Various Environments.

Algorithm	Map Information	Computation Time
RFA-Star	50m×50m×15m obstacle density:0.8	4.1(s)
RRT+DQN (Castro et al., 2023)	300 m³ area with 10 dynamic obstacles	8.2(ms)
Reinforcement Learning (Q-learning) (Souto et al., 2023)	30m×30m with 49 dynamic obstacles	5.67 - 37.43(s)
RFA-Star	90m×90m×15m obstacle density:0.8	9.17(s)
Bionic 3D Path Planning Algorithm (Xu et al., 2024)	100m×100m×100m with 7 dynamic obstacles	148.99(s)
Ant Colony Algorithm (Xu et al., 2024)	100m×100m×100m with 7 dynamic obstacles	59.52(s)
Artificial Colony Algorithm (Xu et al., 2024)	100m×100m×100m with 7 dynamic obstacles	44.67(s)

The RFA-Star algorithm integrates the enhanced A-Star algorithm with the R5DOS model and incorporates a feature attention mechanism. Despite the addition of extra computational steps, its time complexity remains at O(nlog(n)) , demonstrating high computational efficiency and stability in high-density obstacle environments. In contrast, the RRT+DQN algorithm combines Rapidly-exploring Random Trees (RRT) with Deep Q-Network (DQN), making it suitable for path planning in dynamic environments. Its time complexity is O(nlog(n)) , and it shows better flexibility when handling dynamic obstacles.

The Q-learning-based energy-efficient path planning algorithm primarily focuses on reducing computational complexity to extend UAV mission life, with a time complexity of O(m·n) , where mmm is the number of states and nnn is the number of actions. This method is appropriate for scenarios requiring high energy efficiency. The bionic 3D path planning algorithm, which simulates krill swarm behavior, achieves dual optimization of path length and energy consumption. Although its time complexity is relatively high (approximately $O\left(n^{2}\right)$ , it provides superior path planning and energy management in complex terrains.

In contrast, the RFA-Star algorithm consistently demonstrates shorter computation times across various obstacle densities, underscoring its efficiency in handling complex environments. Especially in high-density obstacle scenarios, the RFA-Star algorithm not only maintains a rapid computation speed but also successfully generates stable, safe, and shorter paths, further confirming its exceptional performance in complex settings. Although RFA-Star excels in computation speed, the potential advantages of the RRT+DQN method in dynamic obstacle scenarios should not be overlooked, providing a direction for future improvements in the adaptability of RFA-Star. Additionally, while RFA-Star may not directly compete with the bionic 3D algorithm in terms of energy and path length optimization, its rapid computation and pathfinding capabilities in highly complex environments showcase its robust and stable solution.

## 5 Conclusions

To address the issue of high obstacle density that UAVs may encounter when collecting plant phenotypic information at ultra-low altitudes. Based on spatial topological relationships, this paper introduces the RFA-Star algorithm by incorporating a feature attention mechanism to enhance the A-Star algorithm, providing rapid and stable path planning services for UAVs in high-density obstacle environments. In the path search process, the obstacles are categorized into two situations using condition A, and the feature attention mechanism is introduced to search for characteristic points, guiding the UAV safely to its destination. The study compares the RFA-Star algorithm with RJA-Star, Improved A-Star and A-Star to validate its effectiveness. Experimental results indicate that the RFA-Star algorithm has the shortest computation time, approximately 84%-94% of the RJA-Star and 51%-96% of the Improved A-Star algorithm. The flight distance is comparable to the RJA-Star algorithm, with slightly more explored nodes than the RJA-Star algorithm. Overall, the RFA-Star algorithm exhibits relatively superior performance in terms of computational efficiency and a balanced trade-off between path quality and efficiency. It demonstrates efficient and stable performance in diverse complex environments. However, further optimization is still required to enhance overall performance.

In future work, we plan to enhance the R5DOS model by introducing the more boundary-sensitive RCC8 model. This improvement involves initiating obstacle avoidance when the UAV detects the boundary satisfying the tangent condition with obstacles, ensuring further safety. Additionally, this study was conducted in a static environment; however, in future research, we will consider further refining our algorithm in dynamic and unknown environments. Finally, future work will involve applying the proposed algorithm to real-world scenarios for testing and evaluation, to further confirm its efficiency, safety, and effectiveness.

## Data Availability

The raw data supporting the conclusions of this article will be made available by the authors, without undue reservation.
